# Craniometric determinants of the fitted filtration efficiency of disposable masks

**DOI:** 10.3389/fpubh.2024.1444411

**Published:** 2024-08-20

**Authors:** Jacob S. Griffin, E. Melissa McInroe, Edward R. Pennington, William Steinhardt, Hao Chen, Steven E. Prince, James M. Samet

**Affiliations:** ^1^Oak Ridge Institute for Science Education, Oak Ridge, TN, United States; ^2^Public Health and Integrated Toxicology Division, Center for Public Health and Environmental Assessment, Office of Research and Development, U.S. Environmental Protection Agency, Research Triangle Park, NC, United States; ^3^Department of Occupational and Environmental Health, School of Public Health, Guangxi Medical University, Nanning, China; ^4^Public Health and Environmental Systems Division, Center for Public Health and Environmental Assessment, Office of Research and Development, U.S. Environmental Protection Agency, Research Triangle Park, NC, United States

**Keywords:** face masks, craniometrics, COVID-19, wildfire smoke, public health, mask modification

## Abstract

**Introduction:**

Exposure to harmful aerosols is of increasing public health concern due to the SARS-CoV-2 pandemic and wildland fires. These events have prompted risk reduction behaviors, notably the use of disposable respiratory protection. This project investigated whether craniofacial morphology impacts the efficiency of disposable masks (N95, KN95, surgical masks, KF94) most often worn by the public to protect against toxic and infectious aerosols. This project was registered with ClinicaltTrials.gov (NCT05388201; registration May 18, 2022).

**Methods:**

One-hundred participants (50 men, 50 women) visited the Environmental Protection Agency’s Human Studies Facility in Chapel Hill, NC between 2022-2023. Craniometrics and 3D scans were used to separate participants into four clusters. Boosting and elastic net regression yielded five measurements (bizygomatic breadth, nose length, bizygomatic nasal arc, neck circumference, ear breadth) that were the best predictors of filtration efficiency based on overall model fit. Fitted filtration efficiency was quantified for each mask at baseline and when tightened using an ear-loop clip.

**Results:**

The mean unmodified mask performance ranged from 55.3% (15.7%) in the large KF94 to 69.5% (12.3%) in the KN95. Modified performance ranged from 66.3% (9.4%) in the surgical to 80.7% (12.0%) in the KN95. Clusters with larger face width and neck circumference had higher unmodified mask efficiency. Larger nose gap area and nose length decreased modified mask performance.

**Discussion:**

We identify face width, nose size, nose shape, neck circumference, and ear breadth as specific features that modulate disposable mask fit in both unmodified and modified conditions. This information can optimize guidance on respiratory protection afforded by disposable ear-loop masks.

## 1 Introduction

Exposure to harmful aerosols is of increasing public health concern due to the SARS-CoV-2 pandemic and wildland fires. These events have prompted the public to engage in more risk reduction behaviors, notably the use of disposable respiratory protection which are the primary form of respiratory protection worn by the public against infectious and hazardous aerosols. While disposable masks are commonly worn in countries chronically afflicted with poor air quality, their use in Western countries has increased only recently due to the onset of the COVID-19 pandemic and the growing threats posed by wildland fire smoke (1–3). Access to mask fit testing, however, is limited to N95 respirators used in occupational settings. This lack of mask testing leaves the public essentially uninformed regarding the efficacy of their respiratory protection. Instead, members of the public rely on subjective factors such as cost, availability, and comfort, turning most often to looser fitting, ear-loop style disposable face masks (4).

Studies have shown substantial variation in fitted filtration efficiency (FFE) of disposable masks even after controlling for factors including facial hair and prolonged wear (5–8). This suggests that the heterogeneity in FFE of disposable respiratory protection is at least partially determined by other extrinsic factors that affect the integrity of the seal that is achieved along the mask’s margins. Previous studies have described the relationship between the FFE of respirators and craniofacial morphology and found that protection is a product of the interaction between the respirator and the wearer’s own facial features (9–16). The influence of head and face characteristics on the fitted performance of half-and-full face respirators is unsurprising considering the known human morphological variation that exists in craniofacial features within populations (17–20). Previous studies have found that within population variation in craniofacial morphology is greatest around the nasal and zygomatic bones (17). Given the large amount of variation in size, structure, and shape of the nose and cheeks, these features may be two potential points of leaks that contribute to poor mask performance. In a sample of 73 participants, Oestenstad and colleagues (21) found that 89% of all leaks in respirators occurred at the nose or chin due to the mask not conforming to these areas. O’Kelly and colleagues also attributed individual facial features, particularly prominent noses, as a possible source of gap enlargement when implementing various mask modifications (22). These authors, however, did not quantify the size of the participant’s noses or the size of the visible gaps.

Previous studies on the impact of craniofacial morphology on the FFE of occupational respirators have focused on dimensions of overall face and head size. They have found that measurements including: bitragion-menton arc, bigonial breadth, and bizygomatic breadth all influence the FFE of half-and-full-face respirators, thus further linking overall head and face size to fit and protection (9, 16, 23). While increased head size is advantageous in most respirators due to the straps securing behind the head, this is not the case for disposable ear-loop masks where the ears are the anchoring point where the mask is secured. Though these studies have shown that heterogeneity in craniofacial morphology impacts the fit of respirators, virtually no work has been reported that quantifies the impact of these same anatomical traits on the FFE of the disposable, ear-loop style face masks worn most often by the public during air quality emergencies, specifically KN95, surgical/procedure masks, and KF94 masks (4).

Therefore, expanding from occupational settings to protecting the public at large against the effects of infectious and hazardous aerosols requires consideration of the role of craniofacial morphology as a determinant of disposable ear-loop mask performance. Additionally, the traditional anthropometric techniques utilized in other studies fail to capture the more complex craniofacial structure, particularly mid-facial features, that may lead to points of leakage. Quantifying the impact of the mid-facial structures and its ability to create gaps along the wire nose-bridge of disposable masks requires a new methodological approach beyond that employed in traditional craniometrics. By combining traditional anthropological techniques and a novel mid-face measurement obtained through 3D imaging, we investigated the relationship between specific craniofacial dimensions and the FFE of commonly available masks in 100 volunteers. Statistical clustering techniques separated participants into groups based on a subset of these measurements. In this report we describe the characteristics of four distinct clusters that determine the variation in efficacy of disposable ear-loop face masks.

## 2 Methods

### 2.1 Subjects

One-hundred participants (50 men, 50 women) were recruited for a study at the Environmental Protection Agency’s Human Studies Facility in Chapel Hill, NC. Participants were excluded based on BMI (<19.0, >33.5), blood pressure (≥140 mmHg systolic, ≥90 diastolic mmHg), and history of cardiometabolic or chronic respiratory disease. All participants were non-smokers and had recently shaved (7). Average age and body mass index (BMI) for the final study population were 32.2 years and 25.6 kg/m^2^, respectively. The study was reviewed and approved by the University of North Carolina at Chapel Hill Institutional Review Board and the U.S. Environmental Protection Agency Human Subjects Safety Review Officer.

### 2.2 Anthropometric data collection

A trained biological anthropologist (JG) collected 15 craniometric dimensions and neck circumference using a sliding caliper (Mitutoyo America Corp., Aurora, IL), spreading caliper (GPM Instruments, Zurich, Switzerland), and steel measuring tape (Lufkin, Cooper Tools, Apex, NC) from all 100 participants. Data collection followed the anthropological techniques outlined in Gordon et al. (24) and the data collection protocol created for this study (Supplementary Tables S1–S3). Participants were asked to remove any jewelry that would interfere with measurement collection and to tightly pull their hair behind their head. Each dimension was taken twice and any measurement that fell outside the allowable intraobserver error was recorded a third time. The two closest measurements were then averaged and recorded for data analysis.

### 2.3 3D imaging

3D images were captured using a Bellus3D ARC-7 system (Bellus3D Inc., Lilburn, GA) from participants to analyze craniofacial morphology unattainable using traditional methods. The Bellus3D is a multi-camera system that renders a virtual scan of the subject’s head. Ninety-nine of the images were of sufficient quality for analysis. The 3D mesh produced by the Bellus3D system was analyzed to measure the participant’s nose gap area (NGAP), defined as the area between the nose and anterior projection of the zygomatic bones (see Supplementary Figure S1). This measurement was developed to quantify facial morphology that may be important in determining whether a disposable mask’s wire nose piece can maintain contact with the face.

### 2.4 Quantitative fit testing

Mask fit testing was performed in a 7 × 7.5 ft. stainless steel chamber. A TSI 8026 particle generator (TSI, Shoreview, MN) was used to generate sodium chloride (mean aerodynamic diameter 0.05 microns) particles within the exposure chamber’s atmosphere. The chamber temperature was maintained at 20–25°C and a humidifier was used to maintain approximately 50% relative humidity to standardize conditions and particle movement and deposition during fit testing procedures. Temperature and relative humidity within the chamber were monitored throughout the entire fit test with average temperature and humidity of 23.3°C ± 1.2 and 50.5 ± 4.6, respectively across all 100 subjects. In addition, the chamber had no ventilation to ensure consistent ambient particle concentrations within the chamber’s atmosphere across the testing procedure. Masks were fitted with an aluminum port connected via conductive tubing to a condensation particle counter (CPC—TSI model 3,775) that monitored particle counts/cc in the space behind the mask, while another counter sampled chamber air with 1 s resolution, as previously described (5, 6). Participants were instructed to hold the line monitoring ambient particle counts in close proximity to line monitoring particle counts behind the mask.

Participants completed a modified version of the OSHA Quantitative fit test (CFR 1910.134, Appendix A, Table A2) wearing the following masks: a tri-fold 3M N95 Aura 9205+ (3M, St. Paul, MN), a KN95 (Zhongshan Saifute Labor Protective Articles Co., Guangdong, China), a 3-ply surgical mask (Hannah Linen, Portland, OR), and size large and medium KF94 (Dr. Puri, KM Corporation, Gyeonggi-do, Republic of Korea). The medium KF94 was optional and tested in only 92. While wearing each of the five masks, subjects were instructed to perform the following exercises: slowly bending over to 90 degrees and returning upright for 50 s, reading a standard passage out loud for 30 s, moving the head from side to side for 30 s, and moving the head up and down for 30 s (see Supplementary Figure S2). In addition to the unmodified baseline fit, the effect of tightening the ear-loop straps (KN95, surgical, large KF94, medium KF94) was tested by attaching a clip that tensioned the straps behind the head. A study team member inspected unmodified (without clip) and modified (with clip) masks to ensure each was properly fitted prior to testing. This included showing the participant an instructional video on how to properly mold the metal wire to the nose and guiding them through performing a fit check to test for leaks in the N95. The concentration of particles behind the mask was divided by the ambient chamber concentrations for each one-second interval. The overall FFE was calculated as the average across the entire testing procedure:


$$FFE=100\times \left[1-\left(\begin{array}{l} \mathrm{particle concentration behind mask}/\\ \mathrm{ambient particle concentration} \end{array}\right)\right]$$

### 2.5 Statistical analysis

#### 2.5.1 Dimension reduction

Boosting and elastic net regression methods were first used to reduce the 18 testing variables (age, nose gap area, neck circumference, and the 15 craniometric dimensions). Variables selected for the final analysis were based on their influence on FFE across the four ear-loop masks and lack of correlation to one another to provide the overall best cluster delineation. The FFE of the N95 respirator was not used for the initial variable selection due to the relative lack of influence that craniometric variation has on its FFE due to its high performance and small standard deviations (see Supplementary Figure S3) (25). All statistical analyses were performed in R Statistical Software (v4.2.2; R Core Team 2021) (Supplementary Table S4).

#### 2.5.2 Clustering by craniofacial morphology

Participants were separated into clusters based on shared morphological characteristics using *k*-means clustering which sorts observations to minimize within-cluster variation. Only the variables found during the dimension reduction process were used in the clustering. We performed a linear regression where cluster is the only predictor of FFE to compare the differences between mean FFE for each cluster for all nine testing conditions (unmodified and modified). We followed the same process to compare the differences in the selected craniofacial features between each cluster. The FFE achieved for each mask was categorized based on the performance benchmarks set by FFP1 and FFP2 European mask material filtration standards for particles over 0.3 μm, 80 and 95%, respectively (26–28). In addition, FFE was also compared to a protection level of 65%, a 15% increment below the FFP1 threshold.

#### 2.5.3 Linear discriminant function analysis

Several statistical learning methods were used to test the ability of the model to correctly predict cluster assignment based on the selected dimensions. Ultimately, linear discriminant function analysis (LDA) was chosen due to reporting the lowest test set error. We then tested the ability of LDA to accurately classify the participants by randomly dividing them into a training set and a test set. Initially, we randomly excluded 20 participants and used the remaining 79 to train the model. Next, we used leave-one-out-cross-validation for a more accurate estimate of the error rate for the prediction model. We also used the energy test to determine that the variables did not violate the multivariate normal distribution assumption.

## 3 Results

### 3.1 Clustering by craniofacial morphology

**Figure 1:**
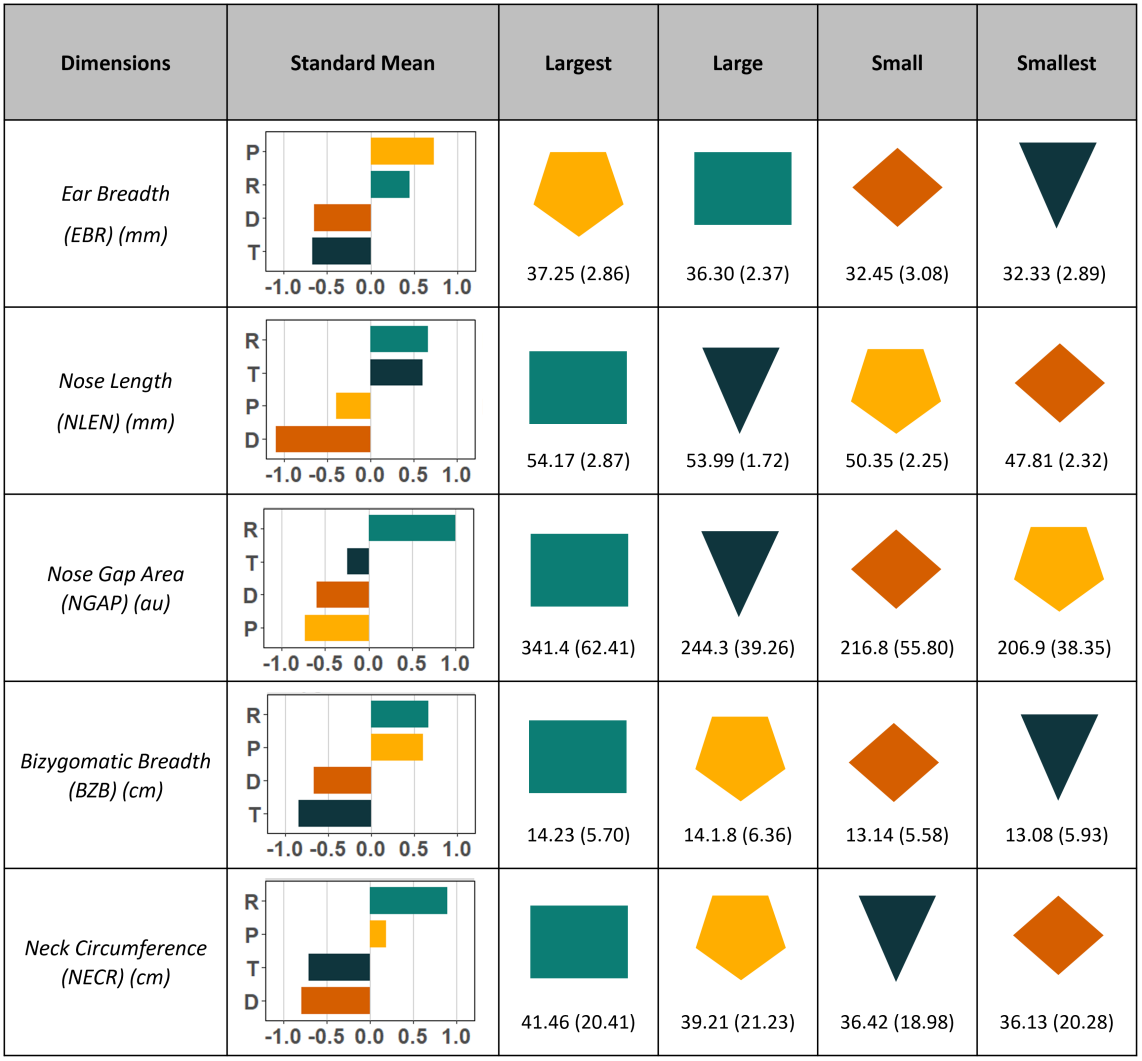
Dimensions selected to separate participants into clusters sorted by their size from largest to smallest. Standardized mean (*z* score) and standard deviation are reported to show relative sizes for each of the five selected craniometric variables used to cluster participants. Nose gap area is reported in arbitrary units. Shapes chosen to represent each cluster act as a visual aide of overall face shape of the cluster. Means (SD) reported from largest to smallest based on four clusters. Cluster rectangle is comprised of individuals with the largest overall dimensions, while cluster diamond is the smallest. Cluster pentagon has small nose length and nose gap area despite having large or largest ear breadth, neck circumference, and bizygomatic breadth. Inversely, Cluster triangle has small or smallest ear breadth, bizygomatic breadth, and neck circumference but large nose length and nose gap area.

**Figure 2:**
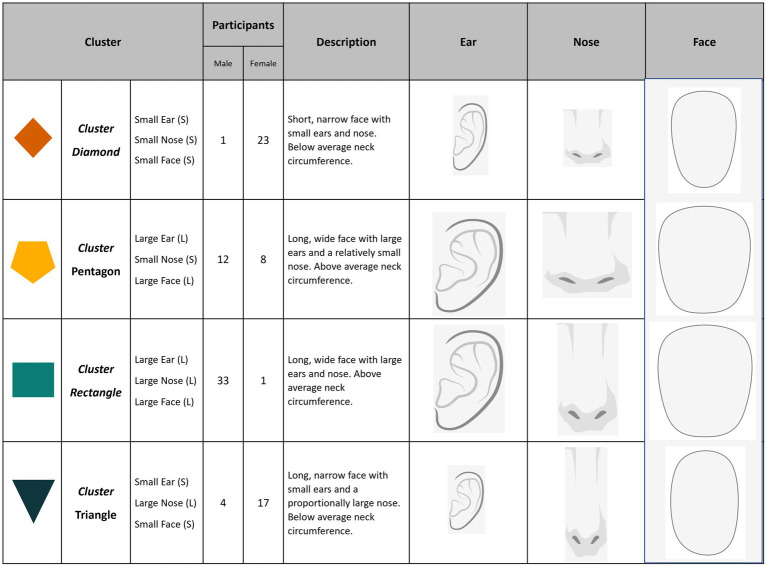
Within cluster visualization, description, and participant count by reported sex. Clusters diamond (95.8%) and triangle (81.0%) are predominately female participants, while cluster rectangle is almost entirely male (97.1%). Cluster pentagon shows the most diversity with 60.0% male and 40.0% female. Overall description of each cluster with images scaled using the group mean and standard mean. Ear images scaled according to ear breath and ear length. Nose images scaled using nose length and nose breadth. Face images scaled using mean bizygomatic breath, bigonial bread, and menton-sellion length of each cluster.

The variables with the most influence on FFE and lowest redundancy across the four disposable ear-loop style masks were identified as nose gap area (NGAP), bizygomatic breadth (BZB), ear breadth (EBR), nose length (NLEN), and neck circumference (NECR) (Figure 1 and Supplementary Table S5). No pairwise Pearson correlation coefficient was found to be statistically significant (*p* < 0.05) between any of these five variables. After using *k*-means clustering with different numbers of groupings, we determined that four clusters provided the best choice based on lower total within-cluster sum of squares, while yielding the most even distribution of participants and a clear separation between them (Supplementary Figure S4). Therefore, these four clusters [assigned as diamond (D), pentagon (P), rectangle (R), triangle (T)] represent individuals with the greatest similarity in overall cranial morphology within cluster, while maximizing heterogeneity across clusters (Figure 2).

Overall, clusters D and T comprise the smallest dimensioned participants, while clusters P and R contain the largest. The major differences between clusters D and T were NLEN and NGAP, with cluster T exhibiting much higher mean values compared to cluster D (Supplementary Table S6 and Supplementary Figure S5). Cluster R has the largest mean values for four of the five selected dimensions. Cluster P is the most evenly divided by sex with 12 men and 8 women, thus representing both small male and large female participants with below average NGAP and above average EBR (Figure 1).

### 3.2 Differences in unmodified mask performance

**Figure 3:**
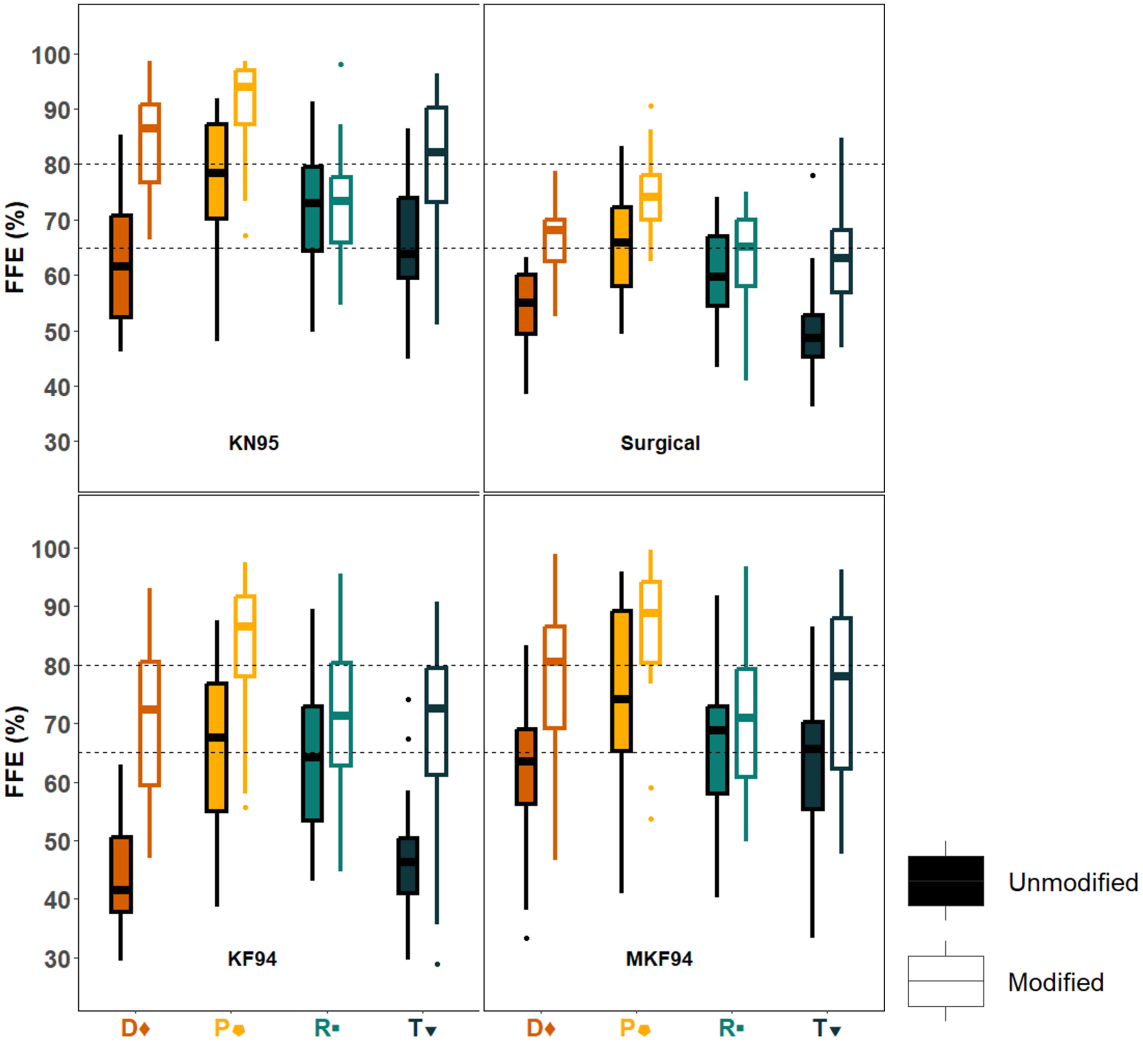
Box and whisker plots of unmodified and modified fitted filtration efficiency (FFE) by cluster for each disposable ear loop mask. The overall FFE for the KN95, surgical, and KF94 masks is shown in box and whisker (interquartile) plots for study participants having performed the modified OSHA Quantitative fit testing protocol. The overall FFE for each face mask was determined by the following expression: [1 −  (mask count/ambient count)] × 100; which is shown as overall FFE (%) plotted against the face mask type tested. KN95 is the best performing disposable ear-loop mask. Cluster pentagon has the highest FFE for all nine testing conditions. Clusters diamond and triangle are the worst performing in all unmodified masks but have the highest improvement when using a clip. Dots above or below the whiskers are flagged as statistical outliers. Dashed lines represent the 65 and 80% FFE protective levels.

**Figure 4:**
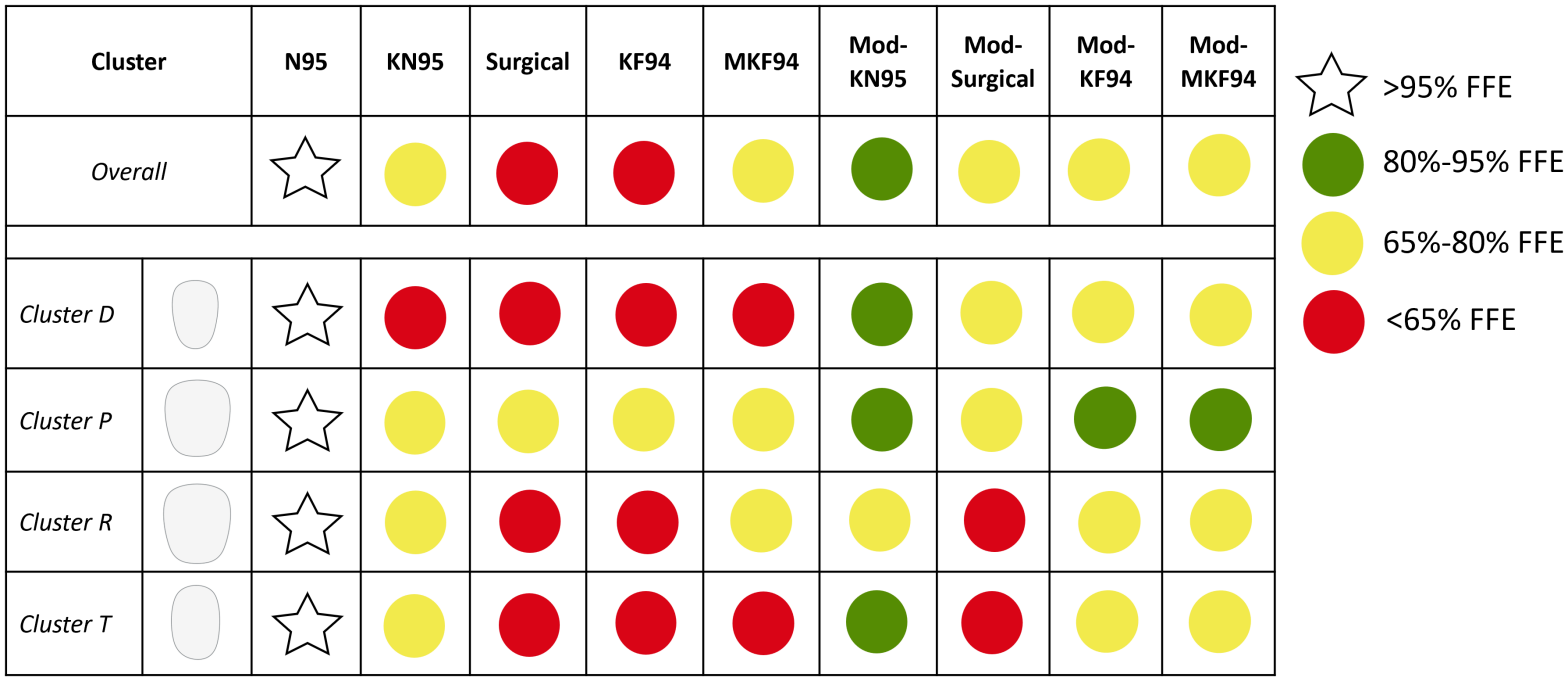
Graphical summary of fitted filtration efficiency (FFE) by cluster. The top row is overall performance across all clusters. In the graphic, green dots are assigned to any mask that achieve a mean FFE of 80% and above. This level corresponds to the European FFP1 mask material filtration standard for particles over 0.3 μm. The level of protection chosen to separate the yellow and red dots was 65% as it represents a threshold which certain clusters could reliably surpass while others only could under certain masking conditions.

The N95 respirator showed relatively little variation in FFE across clusters with mean (SD) efficiencies ranging from 96.8% (4.0%) in cluster D to 98.7% (1.4%) in cluster P (see Supplementary Table S7). The N95 performance exhibited no significant correlation between FFE and the five selected variables (see Supplementary Figure S3). In contrast, considerable differences were found when comparing the FFE of the ear-loop masks between each of the four clusters (Figure 3; Supplementary Figure S6). Overall, the mean (SD) FFE of the unmodified ear-loop masks tested ranged from 55.3% (15.7%) in the large KF94 to 69.5% (12.3%) in the KN95. The KN95 exceeded 65% for all clusters except D (Figure 4). The large KF94 and surgical masks performed the worst with only cluster P reaching 65% in each mask (Figures 3, 4).

Cluster D showed low FFE values for all unmodified ear-loop masks with values ranging from 43.7% (7.0%) in the KF94 to 63.5% (12.8%) in the KN95. Cluster T showed a pattern of protection most similar to cluster D with the FFE ranging from 45.5% (12.4%) in the unmodified KF94 to 66.3% (11.7%) in the unmodified KN95. The surgical was the only unmodified mask where cluster D showed a higher FFE than cluster T [54.2% (7.0%) vs. 49.9% (10.0%)]. Participants in clusters P and R had the best mask performance at baseline with cluster R ranging from 59.6% in the surgical to 71.3% in the KN95. Cluster R were the only participants where the unmodified large KF94 outperformed the surgical mask [63.4% (12.7%) vs. 59.6% (8.2%)]. Overall, participants in cluster P had the best performance for all unmodified ear-loop masks, with FFE ranging from 65.6% (14.7%) in the large KF94 to 77.0% (11.5%) in the unmodified KN95.

### 3.3 Differences in modified mask performance

Mean FFE when tightening the masks with an ear-loop clip ranged from 66.3% (9.4%) in the surgical to 80.7% (12.0%) in the KN95 (Figure 3). The medium and large KF94 masks had mean FFE of 76.4% (14.2%) and 73.02% (14.0%), respectively. The modified KN95 was the only mask to achieve the 80% threshold in multiple clusters. Comparatively, the modified surgical never achieved 80% and only reached 65% in two of the four clusters. With FFE ranging from 63.0% (9.6%) to 72.4% (9.8%), cluster R showed the worst performance across all modified masks, failing to reach 80% in any of the masks and falling short of 65% in the modified surgical mask. Cluster T also failed to achieve 65% in the surgical [62.6% (9.1%)] but had high performance in the modified KN95 [80.8% (11.7%)]. Cluster D never fell below 65% with reported means of 67.0% (6.2%) in the surgical mask and 84.0% (9.4%) in the KN95. Once again, cluster P had the highest FFE for all four modified ear-loop masks ranging from 74.7% (7.1%) in the surgical mask to 90.7% (9.0%) in the KN95. Aside from the N95 respirator, the modified KN95 worn by cluster P is the only masking conditions to achieve greater than 90%.

Cluster D showed the highest degree of improvement when using a clip for all four masks with delta [average (modified FFE − unmodified FFE)] gains ranging from 12.8% in the surgical to 27.1% in the KF94. Conversely, cluster R showed the lowest degree of improvement with gains ranging from 1.1% in the KN95 to 8.2% in the KF94. Compared to cluster R, subjects in cluster P benefitted more from using a clip despite having a higher FFE with unmodified masks.

### 3.4 Linear discriminant function analysis classification rate

Supplementary Figure S4 shows the four distinct clusters plotted on the first and second linear discriminant axes. The initial model tested using 20 randomly selected and discarded participants resulted in an overall correct classification rate of 92.7% (see Supplementary Table S8). All individuals in clusters P and T were correctly identified, while 1/7 (12.5%) participants in cluster D and 1/5 (16.7%) from cluster R were misclassified as cluster P. When using multiple iterations of leave-one-out cross-validation on the initial model, the overall classification rate increased to 97.7% (see Supplementary Table S8).

## 4 Discussion

This study finds that morphological variation in the soft tissue and skeletal structures of the human craniofacial complex is a major determinant of the fitted filtration efficiency (FFE) provided by four commonly worn disposable masks. We identified ear breadth (EBR), bizygomatic breadth (BZB), neck circumference (NECR), nose length (NLEN), and nose gap area (NGAP) as dimensions that were highly correlated with the FFE of KN95, surgical, and KF94 masks, both unmodified and modified with a clip. Specifically, we found that the level of protection that can be achieved when wearing an unmodified or modified disposable ear-loop mask is limited by face width and the size and shape of the nose. Using the five selected variables, we were able to assign participants into four distinct clusters with 97.7% accuracy. Each cluster represents a grouping of participants within the study population that share similar facial characteristics. The ability to accurately classify human faces based on these dimensions and the variation in mask performance between clusters points to the utility of using craniofacial dimensions to predict disposable mask efficiency at an individual level.

Previous studies on the impact of craniofacial morphology on the FFE of occupational respirators found that measurements of overall face and head size including bitragion-menton arc, bigonial breadth, and bizygomatic breadth influence the FFE of half-and-full-face respirators (9, 16, 23). We extended these findings by showing that BZB and NECR significantly influences the protection offered by unmodified and modified disposable masks (9, 10, 16, 23). In addition, we discovered that EBR is an important determinant of FFE that had not been appreciated previously. The benefits of larger head size to FFE has been attributed to the tight seal along the straps at the crown and base of the head in half-and-full face respirators (10, 29). Similarly, we found that ear breadth is an important determinant of FFE in disposable ear-loop masks likely due to the ears being the anchoring point that determines how close a mask fits to the face.

Unlike their larger-dimensioned counterparts, it is notable that cluster D did not reach an average FFE above 65% with any unmodified mask. This indicates that subjects in cluster D were generally mis-sized in the unmodified disposable masks due to their small BZB, NECR, and EBR. Modifying the mask with a clip, however, yielded the largest improvements in protection ranging from 12.8–27.1%. For these individuals with small ear and face sizes, using a clip to secure the ear-loops behind the head likely reduced the size of the gap along the margins of the mask by increasing the tension by the loops. This improved the protection that these masks provide from below 65% to above the 80% upper threshold and illustrates that individuals with smaller faces experience greater benefit from using a clip. This further supports previous findings showing that women, who on average are smaller than men for all dimensions tested, benefit more from mask modifications (29–35).

The influence of larger ears, face width, and overall size was evident in cluster R which had high FFE across all unmodified masks tested. Despite having above average performance at baseline, cluster R participants were the least likely to benefit from using a clip and, notably, failed to achieve 80% protection in any of the masking conditions. This finding is evidence that while larger craniofacial dimensions are associated with better mask FFE at baseline there is an apparent FFE ceiling or “over-tightening effect” that limits performance when modifying the mask with a clip. Cluster R is the most impacted by the over-tightening effect due to having the largest NLEN and NGAP which further limits their potential for FFE improvement.

Other studies have also found that nose shape and size limits mask performance in respirators and when utilizing simple mask modifications or “hacks” (21, 22). In particular, O’Kelly and colleagues (22) noted poorer performance in disposable masks in those with more “prominent noses.”

This suggests that a projecting nasal bridge may lead to large gaps between the nose and cheeks by exceeding the upper limit to which the mask’s wire nose piece can conform (36). This limiting effect of prominent noses is apparent in cluster T where mean FFE is lower than that in cluster D for each modified mask, despite having higher FFE at baseline. The longer nose of participants in cluster T may limit the magnitude of improvement obtained with the clipped condition yielding a smaller increase in FFE compared to cluster D across all masks.

Unsurprisingly, the best protection provided by disposable masks is observed in participants with generally “average” sized craniofacial dimensions. Cluster P subjects had the highest average FFE for every masking condition tested with an FFE that remained above the 65% protection level for each mask tested. This high level of protection is likely attributable to the relatively small noses combined with average to above-average ear, face, and neck size in cluster P. Higher NECR, EBR, and BZB and smaller NLEN and NGAP were shown to be advantageous to FFE across clusters. Cluster P was also the most evenly distributed by sex, with 8 women and 12 men. While previous studies have shown that men are generally better protected in disposable masks than women, the present study shows that both men and women with a specific range of craniofacial dimensions can receive optimal protection from disposable masks (9, 29, 35).

This is the first study to utilize 3D imaging to quantify the nose gap area, a parameter that has only been qualitatively linked to mask performance. In addition, this study employed a modified version of the OSHA Quantitative fit test to generate FFE for four disposable ear loop masks. This test is traditionally only used in respirators, which has limited the information available to the public about the protection level provided by disposable masks. Limitations of this study include the restricted age and BMI ranges of the population that was examined. Expanding the age and BMI range could provide information pertinent to the broader global population given the prevalence of older and/or higher adiposity individuals worldwide. Additionally, only one model of each mask type was tested which may limit the generalizability of the findings. Future studies could test multiple models of commonly used masks to check for consistencies across manufacturers.

## 5 Conclusion

Given the relatively large impact of craniofacial dimension on ear-loop style mask performance identified in this study, these findings provide a basis for improved guidance to the public during air quality emergencies. We identify overall head size, nose size, and ear breadth as specific features that modulate disposable mask fit in both unmodified and modified (clipped) conditions. Specifically, larger faces tend to provide a protective benefit by reducing gaps between the mask and the skin. However, there appears to be a ceiling above which craniofacial size can limit and in some cases impair mask performance. The limiting effect of face and head size was most apparent when modifying the mask with a clip as evident by nose size and shape, which are the most influential factors on whether an individual could achieve 80% or higher in a modified mask.

One-size-fits-all does not mean that one size protects all equally. By defining and quantifying the human variation in craniofacial structure, this study identifies dimensions of the head and face that may lead to guidance for optimizing respiratory protection afforded by disposable ear-loop masks at an individualized level. We show that it is possible to predict the range of fitted performance based on reducing mis-sizing between the face and the mask both for an unmodified mask and when tightening the mask using an ear-loop clip. These findings show that considering the variation in craniofacial dimensions can lead to better public health guidance and optimize the protection that can be achieved with a disposable mask during air quality emergencies.

## Data availability statement

The datasets in this article are publicly available at SciencHub (https://catalog.data.gov/dataset/epa-sciencehub).

## Ethics statement

The studies involving humans were approved by University of North Carolina at Chapel Hill Institutional Review Board and the U.S. Environmental Protection Agency Human Subjects Safety Review Officer. The study was registered with Clinicaltrials.gov NCT05388201 (registration May 18, 2022). The studies were conducted in accordance with the local legislation and institutional requirements. The participants provided their written informed consent to participate in this study.

## Author contributions

JG: Visualization, Writing – review & editing, Writing – original draft, Project administration, Methodology, Investigation, Formal analysis, Data curation. EM: Visualization, Writing – review & editing, Project administration, Methodology, Investigation, Formal analysis, Data curation. EP: Writing – review & editing, Project administration, Methodology, Investigation, Formal analysis, Data curation. WS: Writing – review & editing, Project administration, Methodology, Investigation, Formal analysis, Data curation. HC: Writing – review & editing, Project administration, Methodology, Conceptualization. SP: Writing – review & editing, Supervision, Project administration, Methodology, Investigation, Formal analysis, Data curation, Conceptualization. JS: Writing – review & editing, Writing – original draft, Supervision, Project administration, Methodology, Investigation, Conceptualization.
