# Supplementary material for: Craniometric determinants of the fitted filtration efficiency of disposable masks
Source: Front Public Health. 2024 Aug 20;12:1444411. doi: 10.3389/fpubh.2024.1444411 (PMC11368739; doi:10.3389/fpubh.2024.1444411)
Supplement: Supplementary file 1 [file Table_1.DOCX]

**Supplemental Material**

**
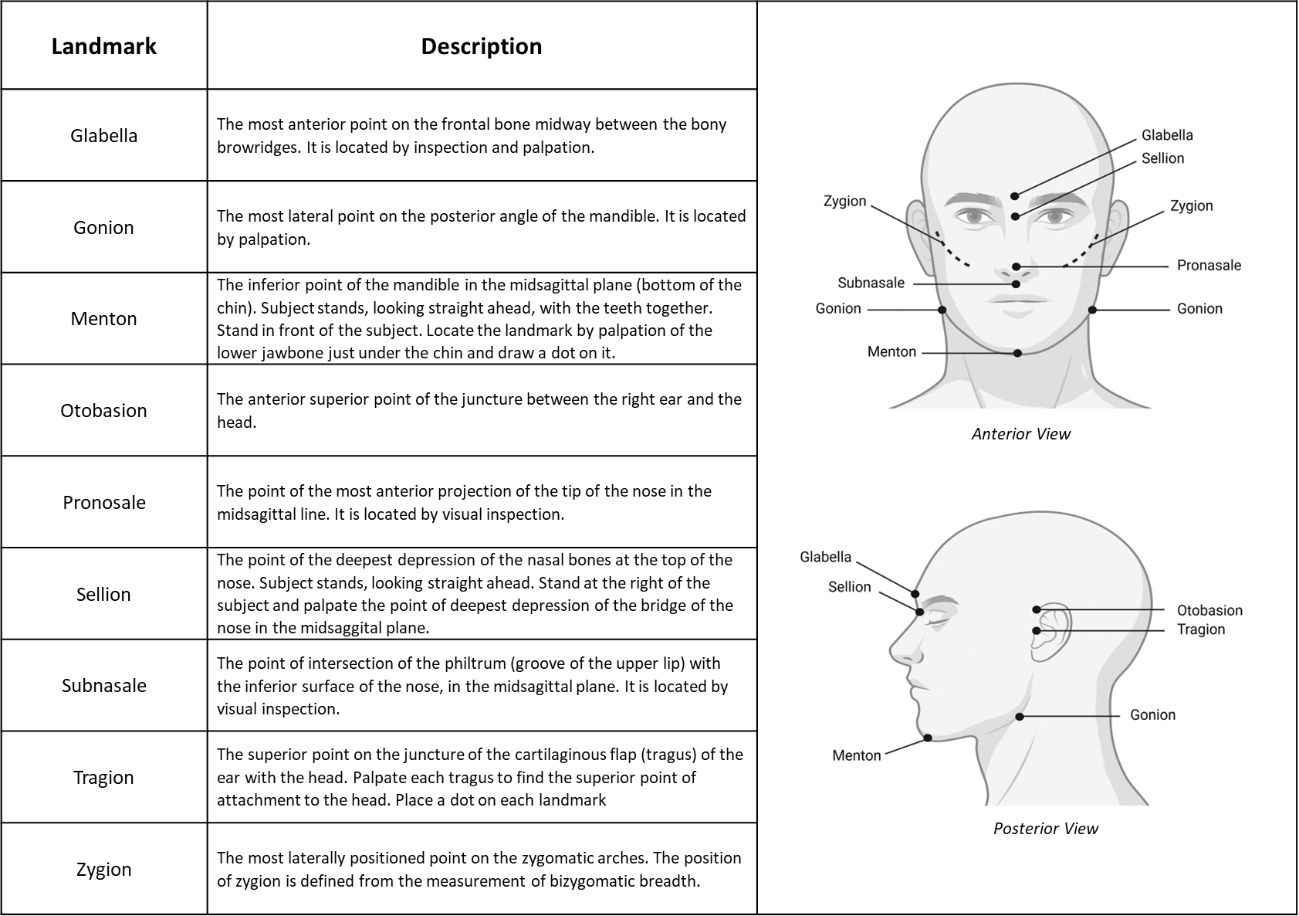
**

*Posterior View*

**Supplemental Table 1. Craniofacial landmarks used to define measurements.** Skeletal and soft tissue landmarks identified on each participant through palpation or measurement prior to data collection.

| **Dimension** | **Code** | **Landmarks** |  |
| --- | --- | --- | --- |
| *Spreading Calipers* | | | |
| Head Breadth | XHB | Euryon-Euryon | The maximum horizontal breadth of the head above the ears measured as from euryon to euryon. |
| Head Length | XHL | Glabella-Opisthocranion | In the midsagittal plane, the distance a point between glabella and the posterior point on the back of the head (opisthocranion). |
| Bigonial Breadth | XGB | Gonion-Gonion | The maximum distance between both gonia. |
| Bizygomatic Breadth | ZYB | Zygion-Zygion | The maximum horizontal breadth of the face between the cheekbones (zygomatic arches). |
| *Sliding Calipers* | | | |
| Ear Breadth | EBR | Otobasion-Posterior Point | The greatest breadth of the ear between otobasion superior and the outside edge of the ear in a line perpendicular to the long axis of the ear. |
| Ear Length | ELN | Superior-Inferior Point | The length of the ear from its highest to lowest points on a line parallel to the long axis of the ear. |
| Lip Length | LLN | Cheilion-Cheilion | The direct distance from cheilion to cheilion as defined as the lateral edge of the mouth |
| Upper Facial Breadth | FBR | Frontotemporale-Frontotemporale | The direct distance between the two frontotemporale points. |
| Face Length | MSL | Menton-Sellion | In the midsagittal plane, the distance between menton at the bottom of the chin and sellion. |
| Nose Breadth | NBR | Alare-Alare | The direct distance from alare to alare as defined as the most lateral edge of the nostrils. |
| Nose Length | NLN | Subnasale-Sellion Length | The direct distance taken perpendicularly between subnasale and sellion. |
| *Tape Measurer* | | | |
| Neck Circumference | NCR |  | Circumference of the neck taken at its base between the manubrium and thyroid cartilage. |
| Head Circumference | HCR |  | The maximum circumference of the head above the supraorbital ridges and ears. |
| Chin Arc | TMT | Otobasion-Menton-Otobasion | The surface distance between the right and left otobasion superior across the anterior point of the chin. |
| Coronal Arc | TBT | Otobasion-Bregma-Otobasion | The surface distance between the right and left otobasion superior across the top of the head in a coronal plane (bregma). |
| Frontal Arc | TST | Otobasion-Otobasion | The surface distance between the right and the left otobasion superior across the forehead above the supraortibal ridge. |

**Supplemental Table 2: Definition of all dimensions recorded during anthropometric evaluation, including 15 craniometric measurements and neck circumference**. Unit of measure and associated landmarks are included for all dimensions.

|  | **Total Sample (n=100)** | | **Male (n=50)** | | **Female (n=50)** | |
| --- | --- | --- | --- | --- | --- | --- |
|  | Mean | SD | Mean | SD | Mean | SD |
| *Chin Arc (cm)* | 33.6 | 20.6 | 35.1 | 12.8 | 32.1 | 15.6 |
| *Coronal Arc (cm)* | 31.9 | 15.4 | 32.4 | 13.2 | 31.4 | 15.3 |
| *Frontal Arc (cm)* | 28.8 | 10.6 | 29.3 | 9.5 | 28.3 | 8.7 |
| *Head Circ. (cm)* | 56.9 | 23.0 | 57.5 | 21.6 | 56.3 | 22.3 |
| *Neck Circ. (cm)* | 38.6 | 30.4 | 40.7 | 24.3 | 36.5 | 20.5 |
| *Ear Breadth (mm)* | 34.74 | 3.50 | 36.36 | 2.94 | 32.99 | 3.17 |
| *Ear Length (mm)* | 63.41 | 5.10 | 66.29 | 3.77 | 60.56 | 4.61 |
| *Lip Length (mm)* | 53.30 | 4.06 | 55.04 | 3.62 | 51.73 | 3.81 |
| *Upper Facial Breadth (mm)* | 104.9 | 4.86 | 106.28 | 4.91 | 103.65 | 4.51 |
| *Face Length (mm)* | 113.8 | 8.23 | 118.8 | 6.69 | 109.1 | 5.07 |
| *Nose Breadth (mm)* | 34.35 | 4.37 | 35.69 | 3.71 | 33.16 | 4.63 |
| *Nose Length (mm)* | 51.84 | 3.60 | 53.19 | 3.21 | 50.42 | 3.46 |
| *Bigonial Breadth (cm)* | 10.5 | 9.1 | 11.0 | 5.9 | 9.9 | 8.5 |
| *Bizygomatic Breadth (cm)* | 13.7 | 8.2 | 14.1 | 6.8 | 13.3 | 6.9 |
| *Head Breadth (cm)* | 15.2 | 6.8 | 15.4 | 6.0 | 15.0 | 6.6 |
| *Head Length (cm)* | 19.4 | 8.3 | 19.9 | 7.7 | 19.0 | 6.4 |

**Supplemental Table 3: Overall mean and standard deviation for each of the 16 anthropometric dimensions.** Means (SD) reported for all 100 participants (50 males, 50 females) and stratified according to sex.

| **Package** | **Version** | **Description** | **Analysis** |
| --- | --- | --- | --- |
| rstatix | 0.7.2 | Pipe-friendly framework for basic statistical tests | Used for correlation analysis to create matrix with correlation and p-values |
| glmnet | 4.1-7 | Lasso and elastic-net regression generalized linear models | Used to perform elastic-net penalized regression to reduce the number variables. |
| gbm | 2.1.8.1 | Generalized boosted regression models | Used to perform boosting to reduce find the relative importance of variables in the model |
| leaps | 3.1 | Regression subset selection | Used to perform best subset selection |
| energy | 1.7-11 | Mutlivariate inference | Mvnorm.etest() to check for multivariate normality |
| MASS | 7.3-58.1 | Support Functions | lda() for linear discriminant analysis |
| emmeans | 1.8.9 | Estimated marginal means (Least-squares means) | Used to calculate the least square means with Tukey adjustments for pairwise comparisons |
| multcomp | 1.4-25 | Simultaneous inference in general parametric models | Used to create the compact letter display of pairwise comparisons |

**Supplemental Table 4: Package name, version, description, and associated analysis for all statistical tests.** All statistical analysis were performed in R Statistical Software (v4.2.2; R Core Team 2021).

| Mask | Boosting (largest relative influence) | Elastic Net (Largest coefficients) | Best Subset Selection |
| --- | --- | --- | --- |
| *KN95* | Ear Breadth 8.86  Lip Length 8.23  Ratio sel.lip 8.12  Nose gap area 8.01  Ear length 6.56  Bizygomatic breadth 5.02 | Nose gap area -2.41  Lip length 1.85  Ear breadth 1.64  Bitragion chin arc 1.26  Neck circumference 0.73  Menton sellion length 0.71 | **Nose gap area -4.83**  Menton sellion length 3.66  **Ear breadth 3.28**  Lip length 3.26 |
| *Surgical* | Bitragion chin arc 13.16  Ratio sel.lip 8.39  Nose gap area 8.29  Bizygomatic breadth 6.25  Mention sellion length 5.02  Ear breadth 4.49 | Bitragion chin arc 2.61  Nose length -2.12  Ear breadth 1.71  Nose breadth 1.71  Menton sellion length 1.60  Bizygomatic breadth 1.55 | Bitragion chin arc 4.71  **Nose gap area -3.26**  **Nose length -3.26**  Menton sellion length 3.19  **Ear breadth 2.55** |
| *KF94* | Menton sellion length 11.38  Neck circumference 10.54  Bitragion chin arc 8.81  Bigonial breadth 6.16  Age 6.13  Nose breadth 4.95 | Age 2.92  Neck circumference 2.47  Menton sellion length 2.18  Bigonial breadth 2.11  Bitragion chin arc 2.01  Ear breadth 1.68 | **Neck circumference 5.18**  Age 5.11  Bitragion chin arc 4.07  **Bizygomatic breadth 3.94** |
| *MKF94* | Bizygomatic breadth 9.80  Nose breadth 7.78  Lip length 7.25  Age 7.23  Nose gap area 6.67  Ratio sel.lip 5.99  Ear length 5.77 | Age 3.42  Ear length -3.03  Neck circumference 2.03  Bitragion chin arc 1.93  Nose gap area -1.92  Lip length 1.83  Bigonial breadth 0.97 | Bitragion chin arc 6.74  Ear length -6.35  Age 5.85  **Neck circumference 4.73**  **Nose gap area -3.59** |
| *All* | Ear length 6.91  Nose breadth 6.75  Ear breadth 6.62  Neck circumference 6.49  Lip length 6.42  Bitragion chin arc 6.23 | Nose gap area 1.39  Bitragion chin arc 1.38  Ear breadth 1.50  Neck circumference 1.08  Menton sellion length 1.01  Bigonial breadth 0.89 | Bitragion chin arc 3.66  **Nose gap area -3.09**  **Ear breadth 2.54**  Menton sellion length 2.27 |

**Supplemental Table 5: Relative influence of each craniometric and age according to clustering method**. Variables selected based on their lack of correlation (redundancy) and overall influence on mask performance. Selected variables= nose gap area, neck circumference, ear length, bizygomatic breadth, and nose length.

| **Cluster Comparison** | **Difference** | **Lower 95%** | **Upper 95%** |
| --- | --- | --- | --- |
| *Ear Breadth (mm)* | | | |
| 2-1* | 4.88 | 2.64 | 7.11 |
| 3-1* | 3.89 | 1.92 | 5.85 |
| 4-1 | -0.07 | -2.27 | 2.13 |
| 3-2 | -0.99 | -3.07 | 1.09 |
| 4-2* | -4.95 | -7.25 | -2.64 |
| 4-3* | -3.96 | -6.00 | -1.91 |
| *Nose Length (mm)* | | | |
| 2-1* | 2.51 | 0.57 | 4.45 |
| 3-1* | 6.35 | 4.65 | 8.06 |
| 4-1* | 6.11 | 4.20 | 8.02 |
| 3-2* | 3.84 | 2.04 | 5.65 |
| 4-2* | 3.60 | 1.60 | 5.60 |
| 4-3 | -0.24 | -2.02 | 1.53 |
| *Nose Gap Area* |  |  |  |
| 2-1* | -10.34 | -52.35 | 31.67 |
| 3-1* | 125.63 | 88.64 | 162.62 |
| 4-1 | 27.79 | -13.67 | 69.25 |
| 3-2* | 135.97 | 96.87 | 175.07 |
| 4-2* | 38.13 | -5.22 | 81.49 |
| 4-3* | -97.84 | -136.35 | -59.33 |
| *Bizygomatic Breadth (cm)* | | | |
| 2-1 | 10.46 | 5.73 | 15.18 |
| 3-1 | 10.94 | 6.77 | 15.10 |
| 4-1 | -1.46 | -6.13 | 3.20 |
| 3-2 | 0.48 | -3.92 | 4.88 |
| 4-2 | -11.92 | -16.80 | -7.05 |
| 4-3 | -12.40 | -16.73 | -8.07 |
| *Neck Circumference (cm)* | | | |
| 2-1* | 29.93 | 13.43 | 46.43 |
| 3-1* | 51.47 | 36.95 | 66.00 |
| 4-1 | 2.68 | -13.60 | 18.97 |
| 3-2 | 21.54 | 6.18 | 36.90 |
| 4-2* | -27.25 | -44.27 | -10.22 |
| 4-3* | -48.79 | -63.91 | -33.66 |

**Supplemental Table 6: The Tukey’s hsd upper and lower 95% CI and p-adjusted value for each pairwise comparison.** Cluster 2 had a statistically higher FFE (p<0.05) than all other clusters in the modified surgical and the modified KF94. Cluster 3 had a lower average FFE, though not statistically different, for all four unmodified masks compared to Cluster 2 with the surgical having the lowest FFE and the KN95 the highest. While Cluster 3 was the second-best performing mask at baseline, it had the lowest FFE in the modified MKF94 and modified KN95 which was statistically lower than the FFE for Clusters 1, 2, and 4.

|  | **N95** | **KN95** | | | **Surgical** | | | **KF94** | | | **MKF94** | | |
| --- | --- | --- | --- | --- | --- | --- | --- | --- | --- | --- | --- | --- | --- |
|  |  | *Baseline* | *Clipped* | *Delta* | *Baseline* | *Clipped* | *Delta* | *Baseline* | *Clipped* | *Delta* | *Baseline* | *Clipped* | *Delta* |
| *Overall* | **97.8 (3.2)** | **69.5 (12.3)** | **80.7 (12.0)** | **11.2* (1.2)** | **57.5 (10.0)** | **66.3 (9.4)** | **8.8* (0.8)** | **55.3 (15.7)** | **73.0 (14.0)** | **17.8* (1.3)** | **66.8 (14.7)** | **76.4 (14.2)** | **9.6* (1.4)** |
| *Cluster D* | 96.8 (4.0) | 63.5 (12.8) | 84.0 (9.4) | 20.6* (1.7) | 54.2 (7.0) | 67.0 (6.2) | 12.8* (1.9) | 43.7 (9.2) | 70.8 (12.6) | 27.1* (2.4) | 62.4 (13.8) | 77.5 (13.7) | 15.1* (3.4) |
| *Cluster P* | 98.7 (1.4) | 77.0 (11.5) | 90.7 (9.0) | 13.8* (2.5) | 65.7 (8.9) | 74.7 (7.1) | 9.1* (1.4) | 65.6 (14.7) | 83.3 (11.7) | 17.7* (2.4) | 74.5 (16.8) | 86.0 (12.6) | 11.6* (3.5) |
| *Cluster R* | 98.1 (3.3) | 71.3 (10.8) | 72.4 (9.8) | 1.1 (1.2) | 59.6 (8.2) | 63.0 (9.6) | 3.5* (1.1) | 63.4 (12.7) | 71.5 (11.7) | 8.2* (1.7) | 66.8 (13.0) | 71.3 (11.4) | 4.5* (1.4) |
| *Cluster*  *T* | 97.6 (2.9) | 66.3 (10.2) | 80.8 (11.7) | 14.5* (1.9) | 49.9 (10.0) | 62.6 (9.1) | 12.7* (1.2) | 45.5 (12.4) | 68.2 (16.7) | 22.7* (2.6) | 63.6 (14.0) | 74.1 (16.0) | 10.5* (2.8) |

*= statistically different at p<0.05 based on paired t-test

**Supplemental Table 7: Overall mean (SD) and cluster mean for each of the nine masking conditions tested.** The mean (SD) FFE of the masks tested ranged from 55.25% (15.66%) to 97.81% (2.16%) with the KF94 having the lowest FFE and the N95 respirator having the highest. Amongst the ear-loop masks, the KN95 was the best performing with a FFE of 69.47% (12.27%). Each of the four ear-loop masks (KN95, surgical, KF94, MKF94) had a statistically significant higher FFE when using a clip to fasten the loops behind the head. The KN95 had the second highest FFE, 80.69% (11.99%), across all testing conditions. While being the lowest overall FFE at baseline, the KF94 had the largest improvement when using a clip (55.25% (15.66%) vs 73.02% (13.99%)). By comparison the surgical mask went from 57.45% (10.00%) to 66.29% (9.38%) and had the lowest FFE when using a clip.

| **Group** | | | **Predicted Group Membership** | | | | **Total** |
| --- | --- | --- | --- | --- | --- | --- | --- |
|  |  |  | **Cluster 1** | **Cluster 2** | **Cluster 3** | **Cluster 4** |  |
| **Original** | **Count** | Cluster 1 | 7 | 1 | 0 | 0 | 8 |
|  |  | Cluster 2 | 0 | 2 | 0 | 0 | 2 |
|  |  | Cluster 3 | 0 | 1 | 5 | 0 | 6 |
|  |  | Cluster 4 | 0 | 0 | 0 | 4 | 4 |
|  | **%** | Cluster 1 | 87.5 | 12.5 | 0 | 0 | 100 |
|  |  | Cluster 2 | 0 | 100 | 0 | 0 | 100 |
|  |  | Cluster 3 | 0 | 16.7 | 83.3 | 0 | 100 |
|  |  | Cluster 4 | 0 | 0 | 0 | 100 | 100 |
| **Cross-Validated** | **Count** | Cluster 1 | 23 | 0 | 0 | 1 | 24 |
|  |  | Cluster 2 | 0 | 19 | 1 | 0 | 20 |
|  |  | Cluster 3 | 0 | 0 | 34 | 0 | 34 |
|  |  | Cluster 4 | 0 | 0 | 0 | 21 | 21 |
|  | **%** | Cluster 1 | 95.8 | 0 | 0 | 4.2 | 100 |
|  |  | Cluster 2 | 0 | 95 | 5 | 0 | 100 |
|  |  | Cluster 3 | 0 | 0 | 100 | 0 | 100 |
|  |  | Cluster 4 | 0 | 0 | 0 | 100 | 100 |

**Supplemental Table 8**: **Linear discriminant function analysis clustering efficiency and reported predictive accuracy for the original model and when using multiple iterations of leave-one-out cross validation.** 92.7% classification rate achieved when leaving 20 randomly selected participants out and 97.7% correct classification when cross-validation.

*
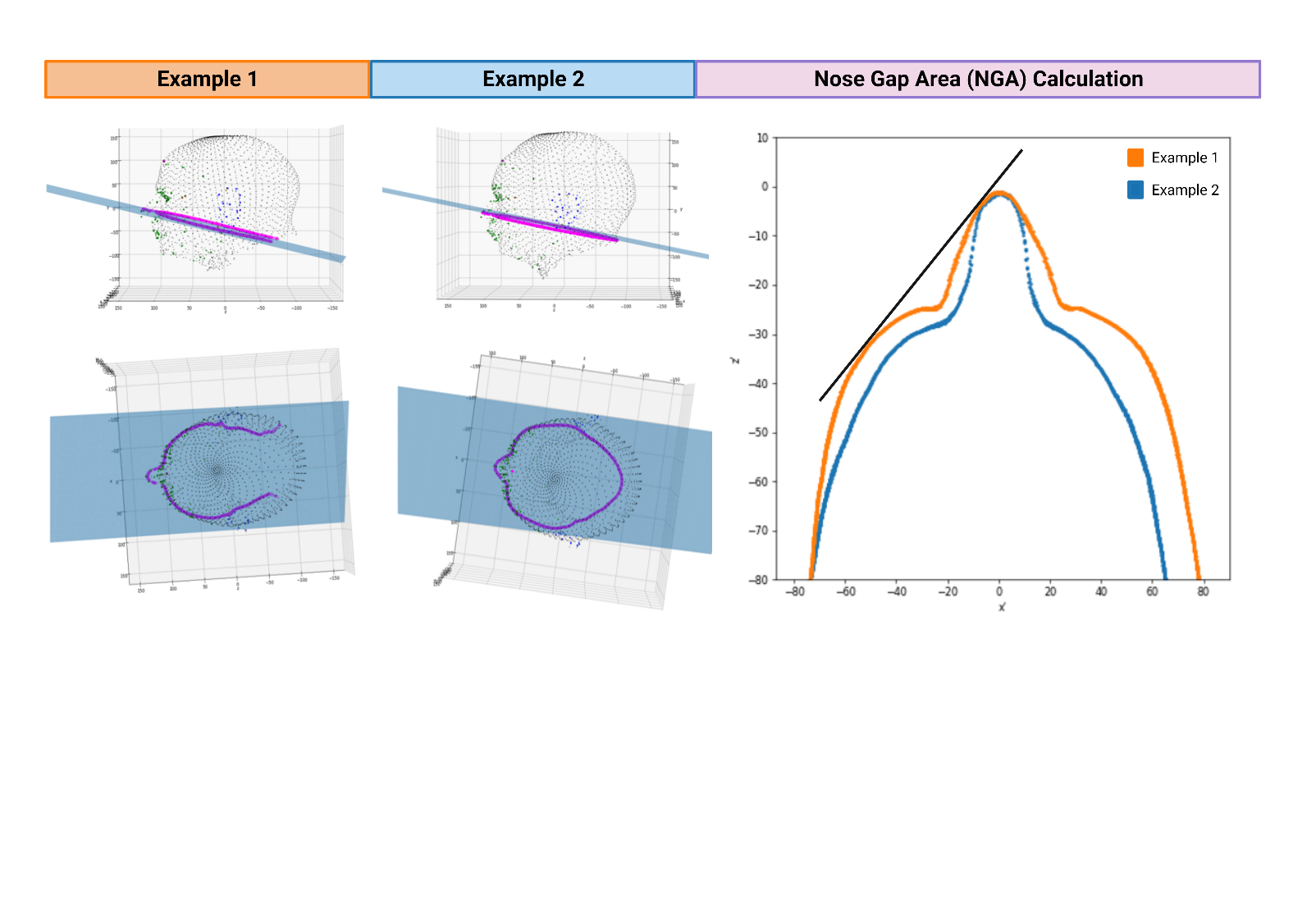
*

**Supplemental Figure 1: Calculating nose gap area (NGAP**). To assess NGAP, the Bellus3D ARC-7 system (Bellus3D Inc., Lilburn, GA) camera mesh data was first extracted and analyzed using the Pandas, Numpy, Scipy, and Matplolib Python libraries. While the imaging software approximates its own craniofacial landmarks via a proprietary algorithm making use of OpenCV, their location was not always reliable due to difficulties in imaging a human face stemming from variation in soft tissue, lighting, and participant orientation. Identifying maxima in a 3D space requires considering the origin of the object in question. In traditional anthropological craniometric data collection, these can be achieved through positioning the head in Frankfort Horizontal by aligning the external auditory meatus and infraorbital margin.

To calculate NGAP, points were selected on the inferior points of each ear and the midpoint of the nose between *sellion* and *pronosale* along the midsagittal plane. The mesh was then rotated so that the z coordinates were in the direction orthogonal to the plane defined by the three landmarks, thus creating a standardized plane across all participants. Points along this plane were extracted, thereby generating a cross-sectional contour along the nose and face. A tangent line was projected onto the contour between the landmark on the nose and the most anterior projection of the zygomatic bones. NGAP was defined as the area between the tangent line and the contour.

With 3D images, however, the same objective can only be achieved after assigning an origin to the mesh. Python functions were written to identify extrema or saddle points in predetermined planes defined by craniofacial landmarks after rotating the mesh within a predefined buffer. Slices were taken from the 3D meshes by defining the contour in question as the points on the mesh within some minimum distance from the plane. This was used to find landmarks on the 3D mesh which could then be used to define the planes in question to quantify NGAP.

**Supplemental Figure 2: Example of fitted filtration efficiency (FFE) with one second resolution for a single participant across the entire modified OSHA fit testing.** The testing procedure for each masking condition consisted of 1) Bending at the waist (50 s.), 2) Reading aloud (30 s.), 3) Looking left and right (30 s.), 4) Looking up and down (30 s.) (x axis). The overall FFE (y axis) is defined as 100 × (1 − behind the mask particle concentration / ambient particle concentration). Overall FFE percentage and SD were calculated across the length of the test. L/R indicates left/right; U/D, up/down.


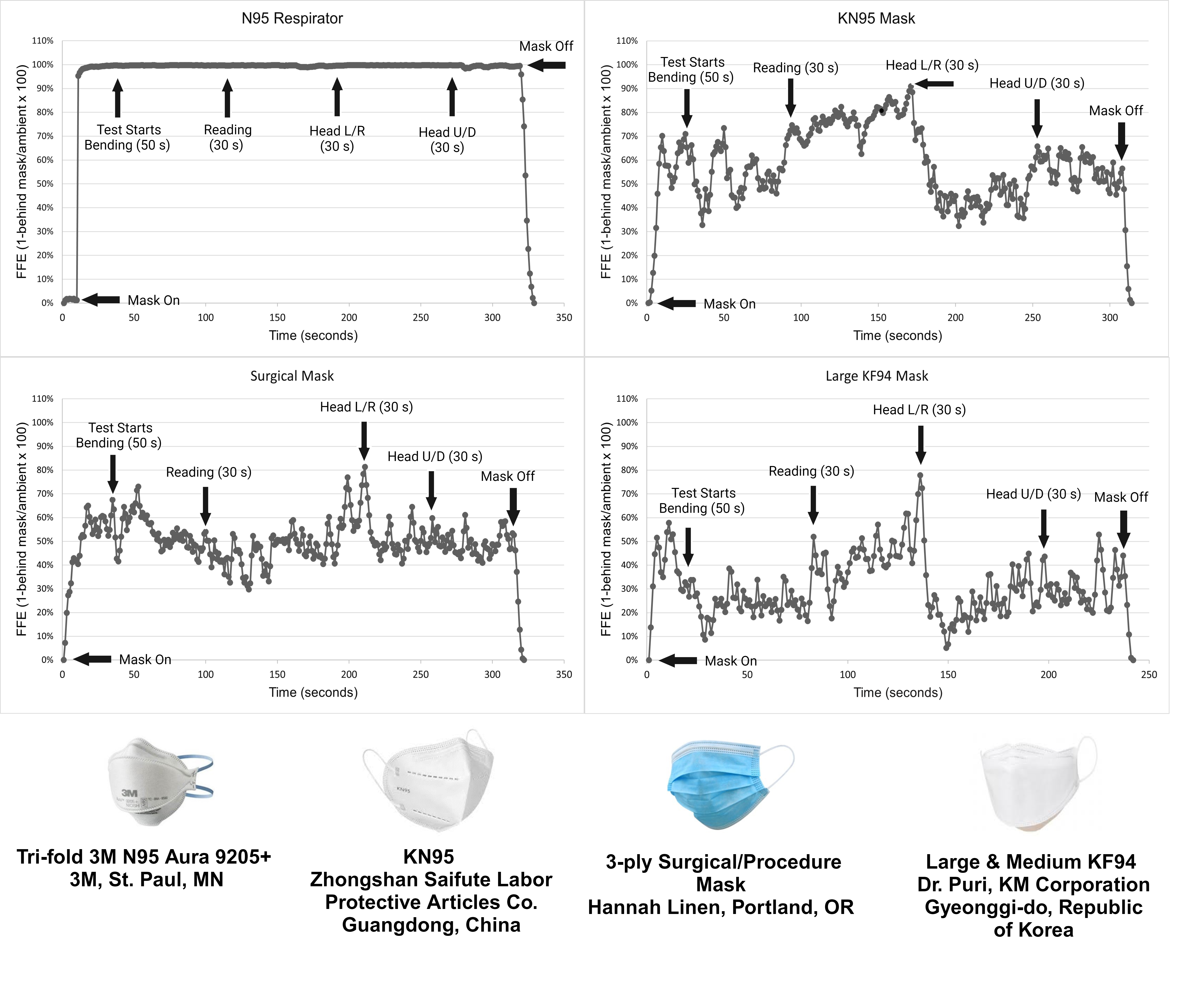


**
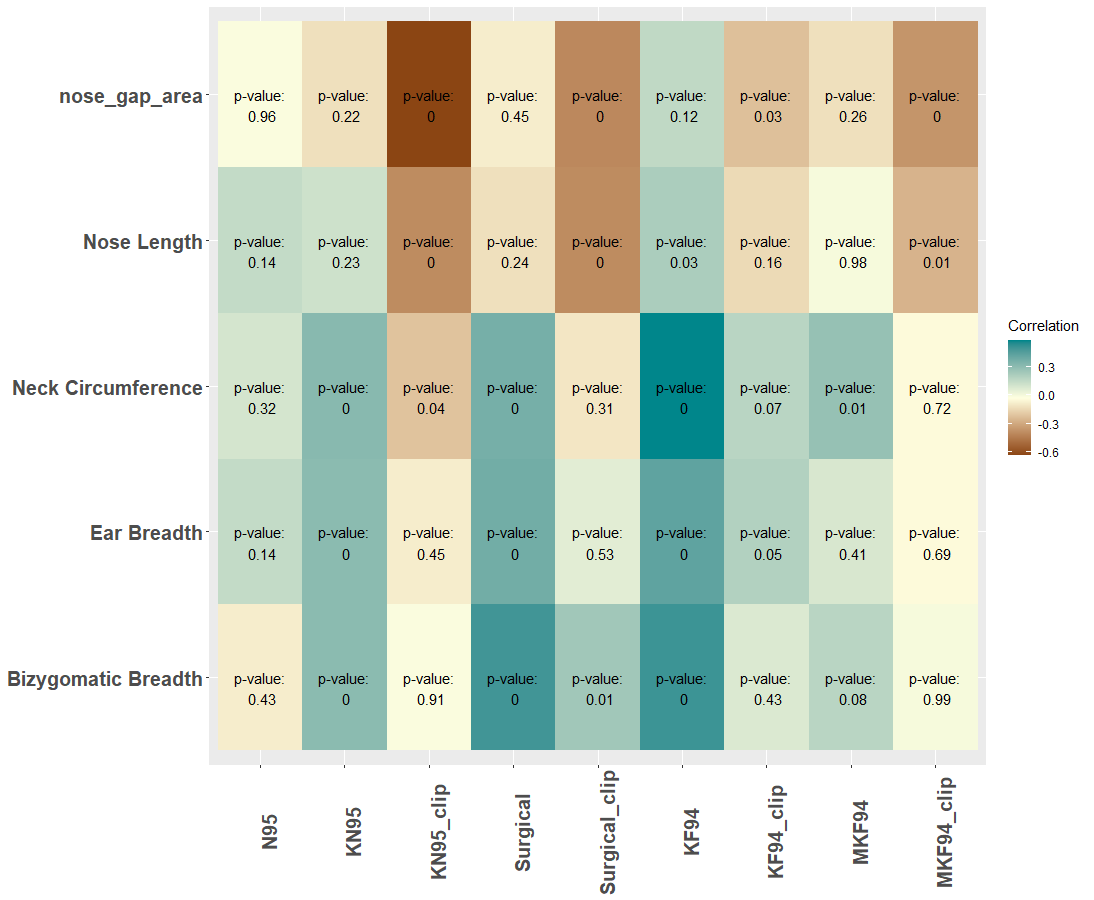
**

**Supplement Figure 3: Heat correlation map showing P value and pearson correlation between each of the selected measurements and mask FFE.** N95 shows no statistically significant correlations to any of the selected variables. Neck circumference, ear breadth, and bizygomatic are positively correlated to unmodified FFE, while nose length and nose gap area are negatively correlated to modified FFE.

**
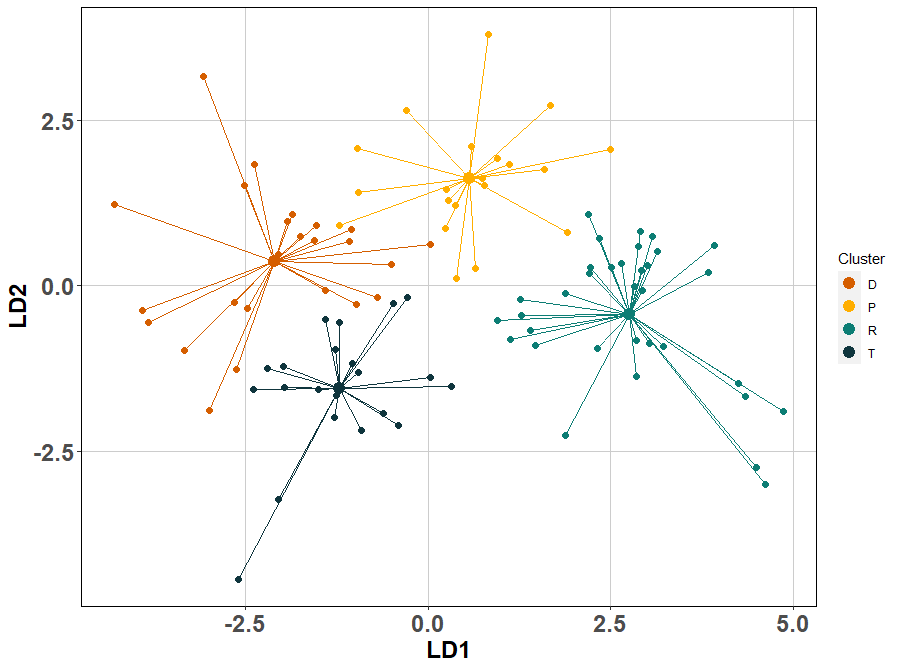
**

**Supplemental Figure 4: Linear discriminant function analysis scatterplot with centroid and all participants plotted on the first and second linear discriminant axis according to cluster assignment.**

**
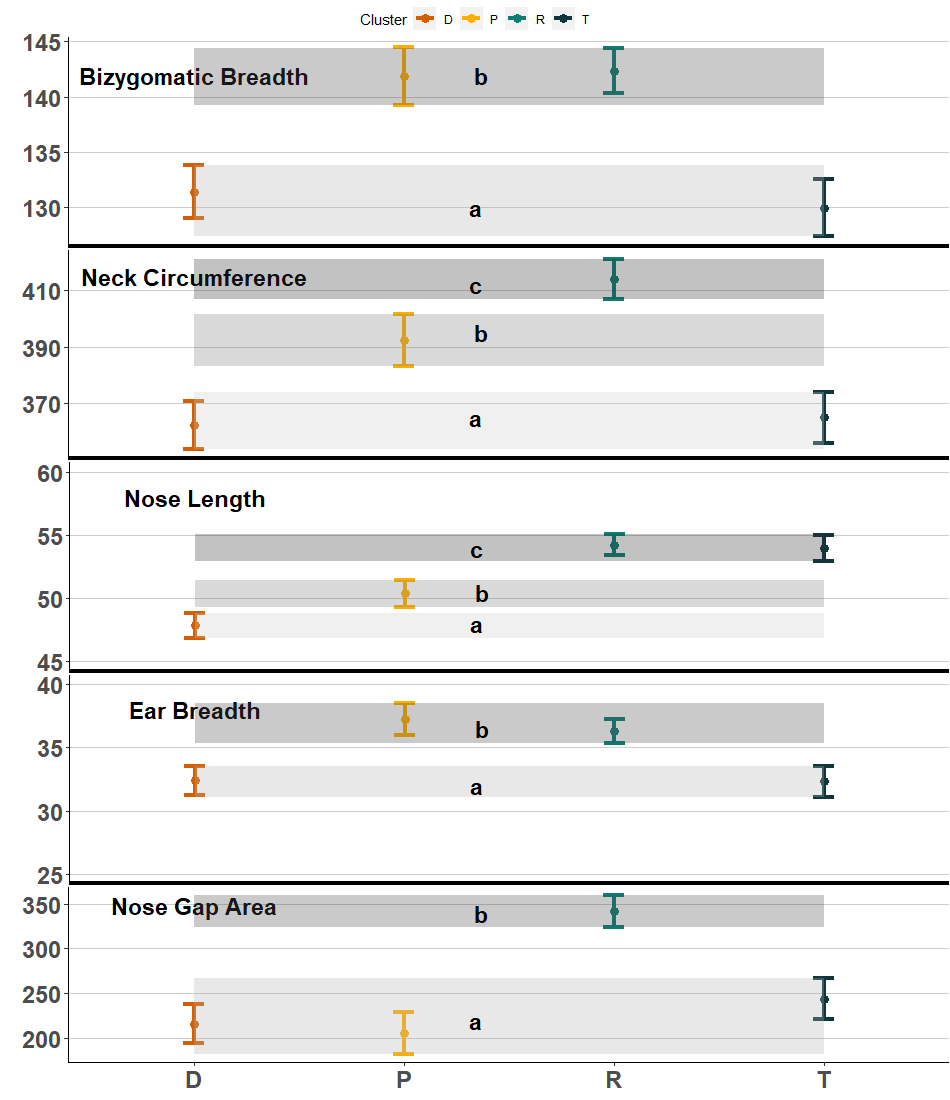
**

**Supplemental Figure 5:** **Post-hoc Tukey’s honest significance difference test (hsd) used to compare the pairwise differences between each cluster. Shapes represent mean with bars indicating first and fourth quartiles.** Cluster 1 mean nose length is statistically smaller than all other clusters (p<0.05) but is not statistically different than Cluster 4 for any of the other four variables. Comparatively, the nose length of Cluster 4 is statistically larger (p<0.05) than both Clusters 1 and 2. Cluster 3 has the individuals with the largest mean for every dimension except ear breadth. Only Cluster 2 has a larger ear breadth though their averages are not statistically different at p<0.05. Neck circumference and nose gap area are statistically larger in Cluster 3 compared to all other clusters.

**
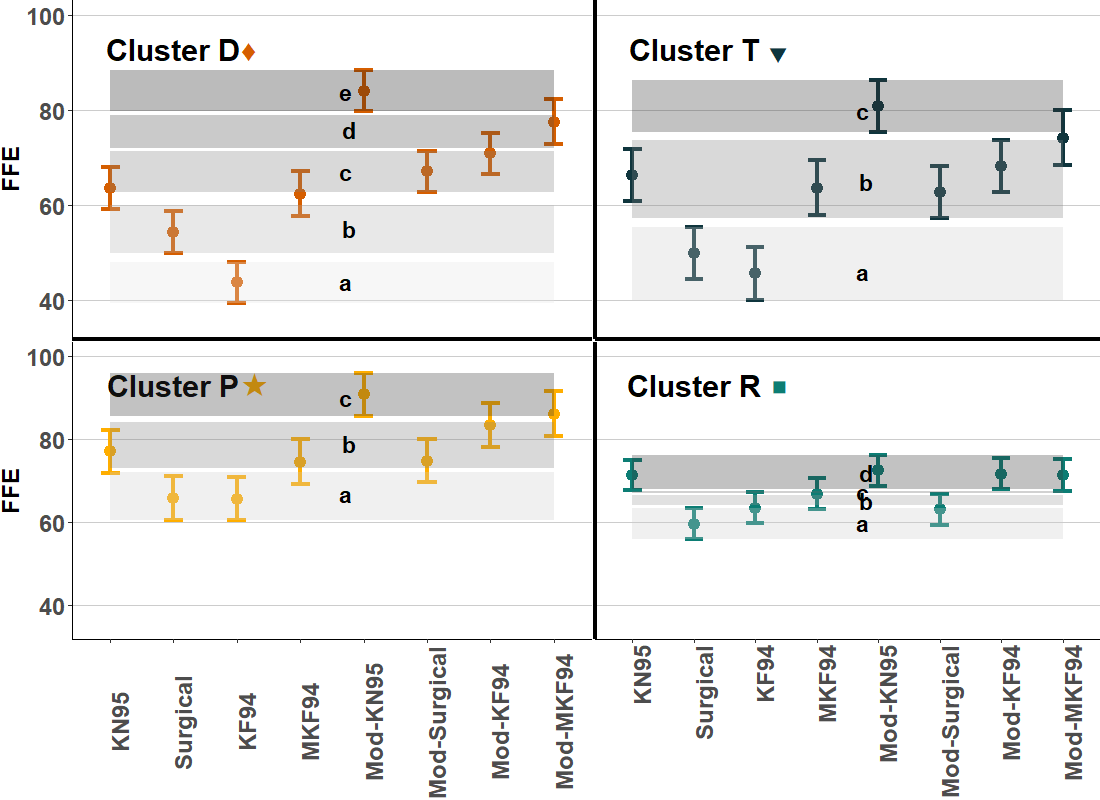
**

**Supplemental Figure 6: Post-hoc Tukey’s honest significance difference test (hsd) used to compare the pairwise differences between each cluster. Shapes represent mean with bars indicating first and fourth quartiles.** Cluster 3 had a lower average FFE, though not statistically different, for all four unmodified masks compared to Cluster 2 with the surgical having the lowest FFE and the KN95 the highest. While Cluster 3 was the second-best performing mask at baseline, it had the lowest FFE in the modified MKF94 and modified KN95 which was statistically lower than the FFE for Clusters 1, 2, and 4.
